# Ezidi voices: The communication of COVID-19 information amongst a refugee community in rural Australia- a qualitative study

**DOI:** 10.1186/s12939-022-01618-3

**Published:** 2022-01-21

**Authors:** Sunita Joann Rebecca Healey, Nafiseh Ghafournia, Peter D Massey, Karinne Andrich, Joy Harrison, Kathryn Taylor, Katarzyna Bolsewicz

**Affiliations:** 1 Multicultural Health Service, HNE Health Harker Building, Wallsend Health Services Longworth Ave, Wallsend, Newcastle, NSW 2287 Australia; 2 College of Medicine and Dentistry James Cook University, QLD 4811 Townsville, Australia; 3 Multicultural Health Service, HNE Health Armidale Community Health Centre, 226 Rusden St, Armidale, NSW 2350 Australia; 4 Central Coast Population Health Unit Level 1, 4 Watt St, Gosford, NSW 2250 Australia; 5 The University of Newcastle, University Drive, Callaghan, Newcastle, NSW 2308 Australia

**Keywords:** COVID-19 messages, Refugees, Ezidi communit, Regional Australia

## Abstract

**Background:**

There is growing evidence that government health information related to COVID-19 has failed to adequately reach culturally and linguistically diverse (CALD) populations in Australia. Refugees are a unique sub-set of the CALD communities and are subject to numerous barriers preventing adequate health care, both pre- and post-migration. The barriers are accentuated during emergencies, such as a pandemic, as a result of an intersection of various social and economic inequalities. The recently resettled Ezidi refugee community in a regional area of Australia is an example of a community sitting at the intersection of various inequities and thus at greater risk from COVID-19. The purpose of this study is to describe the experiences of the Ezidi in a regional area with COVID-19 information and how this has been communicated to and shared within this group; what barriers the community may experience in accessing COVID-19 information; and how the government-led COVID-19 information communication could be improved.

**Methods:**

This qualitative study was designed to explore the perceptions and views of the Ezidi and service providers regarding COVID-19 messaging. Multicultural and Refugee Health staff facilitated interviews with four local service providers and ten Ezidi community members, including seven influential leaders. Thematic analysis was employed across individual, pair and group data analysis. Similar categories were grouped into themes.

**Results:**

The main findings of the study are: the refugee experience influences the communication of COVID-19 messages; cultural, social and gender norms influence responses to COVID-19; trusted individuals and service providers are key in communities’ uptake of COVID-19 messages; currently available governmental COVID-19 information resources and sharing strategies were found unhelpful and inappropriate; COVID-19 communiqués and message delivery for this regional minority refugee community can be improved.

**Conclusion:**

The recently resettled Ezidi community, and likely other similar communities, would benefit from tailored engagement by government organisations, as well as settlement services to improve the communication of COVID-19 health information and reduce related inequities.

**Supplementary Information:**

The online version contains supplementary material available at 10.1186/s12939-022-01618-3.

## Background

While we have all been affected by the tremendous changes as a result of COVID-19, the effect of the pandemic has not been equal across population groups. Globally, the pandemic’s impact has been devastating for some disadvantaged groups [1].Race and ethnicity, together with social and economic conditions, have been shown to heavily influence health inequalities [2]. On almost every health dimension, people of colour have experienced and continue to experience worse health outcomes [3]. Health equity requires that justice is enacted in order to precisely determine how health differences can be fairly addressed [4, 5]. Refugees are a unique sub-set of culturally and linguistically diverse (CALD) communities, who are subject to numerous barriers preventing adequate health care, both pre- and post-migration [6]. Refugees are at a significant health disadvantage compared with non-refugee immigrants due to an intersection of various social and economic inequalities that they face [7, 8]. Recent reports indicate that social isolation, lack of information, stigma and language have all negatively impacted refugee mental health during COVID-19 [9–11].

Difficulties with health care access may also exacerbate the vulnerabilities of refugees during the COVID-19 pandemic in Australia. Refugees may experience barriers to accessing and sharing COVID-19 information, with a trickle-down effect on access to healthcare [12, 13]. In fact, the COVID-19 pandemic in Australia has uncovered the government’s gap in reaching vulnerable CALD groups with suitable health messaging [14–16]. It has been suggested that inadequate government communication with CALD communities contributed to the July 2020 COVID-19 outbreak in social public housing towers in Melbourne [14].

CALD communities exist in all parts of Australia including regional areas of the state of New South Wales (NSW). Armidale is a rural town in NSW, Australia and was designated as a regional refugee settlement location by the Federal Government in August 2017 [17]. It is home to various groups of resettled people including approximately 600 Ezidi. The Ezidi (or Yazidi) are an ancient ethnic minority community who are indigenous to parts of Iraq, Syria and Turkey, many from a rural and impoverished background [18, 19]. Ezidi society follows a hierarchical caste system and marriages are required to remain within the caste [19]. The Ezidi have strong oral traditions, passing on their customs through generations of storytelling in the Kurmanji dialect of the Kurdish language, especially through holy men [17, 19]. Over centuries, the Ezidi people have frequently been the target of persecution, largely due to their exclusive religious and cultural beliefs. Attacks by Islamic State In 2014 saw an estimated 10,000 Ezidi kidnapped or murdered, including women sold into sexual slavery [20]. The Office of the United Nations High Commissioner for Human Rights has termed the killings genocide [17]. The majority of Ezidi still live in Iraq, with smaller communities scattered throughout Europe, North America and Australia [19]. The experience of COVID-19 by smaller refugee communities within regional areas of developed nations such as Australia has not yet been a focus of the disease control effort. If the inequities in disease risk are to change then the experiences and wisdom of communities at higher risk need to be carefully considered.

## Methods

### Aim

The aim of the study was to explore with the Ezidi refugee community in Armidale how COVID-19 information has been communicated to and shared within this group, what barriers the Ezidi community may experience in accessing COVID-19 information, and how the government led COVID-19 information communication could be improved.

### Research design

Qualitative inquiry [21, 22] was chosen to explore and gain rich insights into the perceptions and views of the Ezidi members and service providers regarding COVID-19 messaging. The project was conducted in May 2021 in Armidale at locations familiar to participants, by a multidisciplinary research team comprised of representatives from Multicultural Health, Public Health and academics with expertise in social sciences research. The study received ethics approval from Hunter New England Human Research Ethics Committee: 2020/ETH02955.

### Participants

Three groups of participants were recruited by word-of-mouth, upon recommendation by the Armidale Refugee Health Nurse. These included: 1) local agency service providers who work directly with the Ezidi population in Armidale, such as staff representatives from settlement agencies and educational facilities; 2) influential Ezidi community members, who were mostly elder men and 3) Ezidi women; aged between 20–50 years old. A further five Ezidi men were invited but declined to participate in the research. All Ezidi community member participants, including influential leaders had arrived in Australia after 2016.

### Setting

The study occurred in a regional NSW town during a period of no community transmission of COVID-19, but with occasional hotel breaches in capital cities. During the study period many countries were experiencing significant waves of infection from new variants. Although there were no COVID-19 transmissions amongst the Ezidi in Armidale, the community were aware of the effects of COVID-19 affecting Ezidi family and friends overseas. The roll out of the vaccine program had commenced but only a small proportion of people were fully vaccinated.

### Procedure

The participant information statement for community members was presented as a short video delivered in Kurdish-Kurmanji language prior to obtaining consent. Community members had an opportunity to ask questions about the study, with an on-site Kurdish-Kurmanji interpreter present. Verbal consent was collected, as some community members refused to document a signature on the consent form. Interviewers sensed discomfort from the community members and did not probe further for signatures. Service providers received written information statement and provided a written consent.

We developed a semi-structured interview guide and piloted it with a few Syrian and Congolese refugees. The guide included questions on ways in which the community has received COVID-19 information; what made it difficult; and how access and the use of COVID-19 information could be improved for Ezidi community in Armidale (Interview Guide in Appendix). The interviewers were employees of the Hunter New England (HNE) Multicultural Health Services: two refugee health nurses and a multicultural health liaison officer. One of the nurses was well known and accepted by the community. Prior to commencing data collection interviewers underwent a four-part qualitative training coupled with practice on interviewing, facilitating discussions, using recording equipment and conducting preliminary data analysis.

We conducted three focus group discussions in English: service providers (four), influential community members (six) and community members (three). To minimise power imbalance, we interviewed the influential community members in a separate group to the community members. Kurdish-Kurmanji health interpreters were available on-site to translate discussions with community members. Seeking richer data and clarification of cultural matters, researchers interviewed an additional (English speaking) influential community member by teleconference, approximately three months after the initial focus group discussions. Focus group discussions lasted 30–60 min and were audio-recorded. A native English-speaking member of the research team transcribed verbatim the English components of the collected data.

Senior qualitative researchers provided guidance and oversight for data analysis. Each transcript was read and reviewed separately by two interviewers, each creating an individual analysis file before meeting to pair their analyses. Paired analysis involved discussing and comparing individual analyses and creating a combined analysis file for each transcript [21]. After reviewing several transcripts, similar concepts were grouped into categories [21]. Group meetings were held regularly with senior qualitative researchers to discuss paired analyses, agree on categories and sub-categories and identify further insights. Five overarching themes were identified across transcripts via the iterative group process.

## Results

Five themes were identified

1. The refugee experience influences the communication of COVID-19 messages
2. Cultural, social and gender norms influence responses to COVID-19
3. Trusted individuals and service providers are key
4. Problems with available government COVID-19 information
5. COVID-19 communication can be improved

### The refugee experience influences the communication of COVID-19 messages

Community members and influential community members reported low literacy, not only in English, but also Kurdish-Kurmanji. This was attributed to dislocated education prior to arrival in Australia, lack of opportunity and competing priorities:

*I used to be able to write Kurdish and Arabic as well, but for 6 years [as a refugee] I didn’t write anything, it got lost (community member)*

Older community members stated that they are not familiar with technology; therefore, they relied on younger members to receive COVID-19 messages. Access to technology equipment, such as a device or Internet was reported to be problematic:

*Lots of parents, the thing they wanted most for their kids- ‘teach them about computers!’* (service provider)

*It [COVID-19] did make the community very aware of their lack of digital literacy* (service provider)

Importantly, both the community and service providers reported that psychological issues related to past traumatic experiences thwarted the refugees’ ability to process and learn new information related to COVID-19 information. Underlying mental health issues, which seemed to be related to the refugee experience, were reported to have profound effects on memory and retention. The Ezidi participants struggled to prioritise daily tasks, understand, retain and recall information. An elder Ezidi man described his experience of personal loss associated with the Islamic State attacks, then pointed to his head and said:

…*the brain is tired, so we can’t learn* (influential community member)

One service provider gave an example of the difficulties with processing and retaining health related information by Ezidi community members:

*[Due to the trauma, community members don’t have the ability] to retain and understand, and to put that into real life as well. They might have been told that they can’t go anywhere [after their COVID test], but then the dentist would have called and said, ‘you’re late for your appointment’… so that’s all gone out the window* (service provider)

### Cultural, social and gender norms influence responses to COVID-19

Service providers described how Ezidi socio-cultural norms relating to age and gender influenced the way community members sought and shared COVID-19 messages. For example, although younger men may have desirable attributes such as bilingual ability and networking capability through workplaces, service providers felt that they were hesitant to pass on information without authorisation by respected older males.

*But they’re in a good place [young men working in services], the younger ones- because they have more English and have more access to working culture…. I think in a few years’ time, they will naturally take that mentor up of being those community leaders* (service provider)

*I think there’s a small group of…they are all men, but younger men who have taken up a bit of a community leadership role but at the same time they don’t actually want to be community leaders either* (service provider)

This is in contrast to the report from an Ezidi influential member who said other community members actively sought out information from younger Ezidi influential people:

*They [community members are] usually asking people who are working already with the organisation.... we have got 2 or 3 people who are working with them. So, they ask them. If there is any situation happen in Armidale in particular, we all would be connected with each other* (influential member)

Additionally, service providers thought that information delivered by women might be less acceptable to men. They also believed that women are less likely to actively seek information:

*The women, I think, they are the group who are probably least informed. They are also the group of people who are least likely to go into our schools and to go and do other work. It’s basically the men who do a lot of that* (service provider) 

*[single women] are also the ones who are going to get that information from families back overseas and have that misunderstanding of what’s going on as well, and they’re not going to reach out to services for that information* (service provider)

One female community member reported that she relied on male relatives (uncles and brother) to gather information about COVID-19, as they were literate in Arabic. Another female community member was literate herself and said she actively sought out COVID-19 information. An older male influential member talked about the variable community uptake of messages he and other elders had shared amongst the community through word of mouth. Some older males reported that despite literacy in Arabic, being digitally illiterate and having low proficiency in English meant that accessing COVID-19 information remained difficult. An incomplete understanding of COVID-19 was considered by service providers to be a contributing factor to fear amongst the community. An influential community member commented that news about the developing COVID-19 situation in their homeland contributed to the community’s fear response. During the first wave of COVID-19 infections in NSW, service providers noticed Ezidi people carry out customs and behaviours and attributed them to fear. Some Ezidi people were seen wearing homemade red and white threaded necklaces and bangles, which is a practice traditional to the Kurdistan Ezidi group as an expression of protection and love:

*they were fearful of sending their children to school, even before, when it [COVID-19] first just started coming out. You could see, they were already coming in wearing those sorts of things [red and white threads] (service provider)*

*I actually saw an increase of kids even wearing you know, the threads…the red and white threads. I actually saw an increase in number of people wearing those during COVID* (service provider)

Community members reported people locking doors to protect themselves. Some service providers suggested an alternative interpretation of door shutting.

*March 2020… that [locking doors with chains], that happened in the beginning... like there was a fear in the community because we lost lots of people in our country in Iraq with the fear situation* (influential community member)

*During lockdown a lot of people kept their door shut and their gates shut as well, because they were so scared about the whole situation. Because they just didn’t have the understanding of what this was… they put chains all on their doors and their gates and they padlocked their gates shut so no one could come in or out* (service provider).

Service providers thought that community behaviours were shaped by a historic fear conditioned response to the ISIS raids in their homeland.

*People’s cultural background has had a really significant background on how they have responded. And I think that has a lot to do with what’s happening in their home country, or their home village. Because the experience that their family overseas are having, they feel like they need to respond in a certain way* (service provider)

### Trusted individuals and service providers are key

Community participants received information from a variety of people and platforms they trusted. People with low level of literacy relied on word of mouth from family and friends in the community or overseas. Close relationships within the community, coupled with the benefits of proximity living in a country town, facilitated the spread of COVID-19 messages to community members.

*Because that [information about COVID-19] is from their home country, I think there is a higher degree of trust that comes with the information that comes from that location* (service provider)

*And Armidale is a small town, with everyone visiting each other every day or every week. I guess, so it's easier to share information with other community* (influential community member)

However, most information was received directly from familiar local service providers such as schools, a tertiary education setting (Technical and Further Education- TAFE), settlement services and refugee health nurses. All Ezidi participants reported a deep sense of respect for the trusted sources, especially local service providers. Community members consistently identified the same service providers as key to their understanding of COVID-19 information:

*So they are health professionals- they got lot of information, the right information about it. So, in terms of our caseworkers they care about us, so they have to tell us the right information. So if we do not get from them, where we have to get from?* (influential community member)

*So here I don’t believe in anyone but her [refugee health nurse] ...yeah, we have a lot of respect and trust in her* (community member)

*… if it’s a person that I really trust, and she kind of looked at that [COVID-19] information and given me…I trust her. So I only trust people that I really know. But I really need to know the person….* (community member)

But a mistrust of government:

*…[the Ezidi] have a very big mistrust of government, so they are not necessarily going to go to government sites to find that information. They’re going to go to ‘D’ at school and knock on her door, or they’re going to go to ‘J’ in Health…* (service provider)

Moreover, participants felt that inconsistent and frequently changing COVID-19 messages across Australian states had contributed to the community’s distrust in governmental information:

*… there’s conflicting information out there. And the community becomes a bit paranoid, you know, ‘did I get the true information before’ or ‘should I trust that source, I don’t know’* (service provider)

*…lot of times the information we are given- sometimes it is not right. It’s wrong. That’s why it’s hard to believe it* (community member)

### Problems with available government COVID-19 information

The Service providers and community members reported that the Armidale Ezidi have been assisted by local service providers during the pandemic, who took initiative and filled the void of COVID-19 information reaching the community. The service providers described that they shared COVID-19 information by phone call (through interpreters) and social media. Settlement services translated written information into audio-files and shared them on social media platforms such as WhatsApp and Facebook.

*We use those resources [NSW health website and TV] to make the video and then we post it on the Facebook page* (service provider)

*…there is a link to making your own video, which was probably the most useful thing we had, because I could just give it to some support workers and say ‘please build a video for us’ and they did that and it was great* (service provider)

However, service providers reported feeling under-supported by government and uncertain about the content of information they delivered, particularly at the beginning of pandemic. Service providers added that inconsistent messages contributed to community confusion.

*…[the information] is not consistent but also there’s a lot of it. So, it’s hard to comprehend* (service provider)

*So, using masks for example, was a really good example of how things shifted. You know, for the first while, the government’s advice was don’t wear a mask. You don’t need to wear a mask, unless you’re sick. And then all of a sudden, that switched. And so you’ve just spent all this time educating everyone on, ‘no its ok, you don’t need to wear a mask unless you’re sick’ and then the government advice changed and then we just had a bunch of people who were confused and ‘do I wear a mask? Do I not wear a mask?’* (service provider)

Despite being native English speakers with high literacy, at times even the service providers themselves reported difficulty finding accurate COVID-19 related information from government media sources. Concerns followed that the quality of message disseminated to the Ezidi community could be suboptimal. Service providers criticised government COVID-19 resources for the Ezidi and other CALD communities. They reported that information was not translated into languages and script used by Ezidi community, and that the information was too complex for the literacy level of Ezidi community.

*…the only other important thing when we’re talking about information given to multicultural communities is the simplification of the information, so breaking it down- ‘what is COVID? what is this disease? how is it transmitted?’…. that kind of basic foundational information, is disseminated through the community, by the community* (service provider)

*The problem we now have is that there are now resources in Kurdish-Kurmanji, but they are in the wrong script. So, nobody can read them. And even if they could read them, the vast majority of the community can’t read Kurdish-Kurmanji…they’re illiterate…so it needs to be verbal and …pictures* (service provider)

Another problem identified by service providers was inconsistency and variation of information from each state.

*What really stuck out for me, is how differently each state has managed information. And it needs to have been something that was managed at a federal level. I mean, common sense should prevail that the information going out to the Australian population should be from a central location. Because the advice from different states was different, and so if you were looking to find information to share to the community, you go ‘well is NSW or Queensland more on the topic’, you know, I don’t know* (service provider)

### COVID-19 communication can be improved

Although community members seemed satisfied with the ways they have been receiving COVID-19 information, both service providers and community members discussed that the content, format and delivery of COVID-19 information could be improved to better reach the Ezidi community (Table 1).

**Table 1:** Suggestions for improvement of official COVID-19 information for Ezidi community in Armidale, Australia (2021)

**Participant suggestions for government sharing of official COVID-19 information**
• Listen to community
• Provide clear, consistent and culturally appropriate messages
• Recognise diversity within the ethnic group
E.g., language, religion, literacy ability, English proficiency
• Translate using dialects specific to community groups
• Choose multiple methods to distribute official information
E.g., direct telephone calls, written handouts in language and social media videos
• Identify and consult with key Ezidi contacts
• Delivery via trusted community members and service providers
• Utilise highly frequented and familiar spaces to deliver messages
E.g., TAFE, school, medical and dental clinics, multicultural supermarkets, social media

Service providers reported that COVID-19 related information needed to be orally presented in the Kurdish-Kurmanji dialect. Suggestions were made to use pictures or audio-visual material to present information, or a phone call by trusted service providers. Younger community members suggested also having a form of written material available in language for those who were literate:

*The information here can be given to the community like over the phone, or even like the leaflets, the paper. And my brother or my sister, they can read both languages. But if it’s written- maybe if it’s Arabic, it’s easier for them to understand* (community member)

*Some people don't understand...they not attending school at all...A better way to share the information in regard to COVID-19 to make a video from a person from the Ezidi community then everyone can understand clearly and they can get the message very well* (influential community member)

*I think simple pictorial stuff…Australia is such a multicultural community, that the list of languages is just going to be infinitely long. And if I say, ‘hey, here’s 60 flyers to put in your foyer, so we can cover all the languages’, it’s just so much information that people aren’t going to bother shuffling through that* (service provider)

Service providers felt the content needed to be simpler, clearer, concise and relevant. Consistency in content and a unified message was reported to be important, for both service providers and Ezidi members. In addition, service providers felt that the messages needs to be gender, age, and ethnic specific, in order to be culturally appropriate:

*It [COVID information] needs to come from a federal level, clear and concise, unified information* (service provider)

 *[The information] should have photos of different ethnic backgrounds, so you’ve got something that will actually resonate with them, rather than your Anglo-Saxon...*(service provider)

Another suggestion was to utilise social media platforms such as Facebook and WhatsApp, which are already familiar and trusted by Ezidi people to share information. Service providers suggested audio-link files in language as an easy adjunct to social media or government sites:

*We can even message out [through social media] a small file to all our clients* (service provider)

*WhatsApp particularly, for lots of multicultural communities, that’s the go-to communication device* (service provider)

Furthermore, all participants highlighted that important health messages must be delivered through trusted service providers or community members. Service providers recommended the identification of ‘key Ezidi contacts’, who may not be revered as official community leaders, but do possess desirable attributes, such as being bilingual, educated and connected to people outside the Ezidi community through workplaces. Service providers described that the importance of engaging these individuals is to harness their skills and cultural knowledge, thereby channelling COVID-19 messages in the most culturally appropriate and efficient ways. Service providers predicted that these individuals would likely become accepted as community leaders in the future:

*If you can get someone from our community and they can video….and everyone from our community can listen to the video and share with friends* (influential member)

*I think making it a friendly face...people really didn’t want to talk to people they didn’t know. Finding somebody local to then record that [COVID-19 information audio-visual material], and being able to share that* (service provider)

*…. [a] program, where they train community members and then those community members go out and do group work within the community to spread that message* (service provider)

School and TAFE were identified as avenues to distribute COVID-19 information, as compulsory attendance provides the opportunity for teachers to ensure messages are sent home with a student, thereby potentially benefiting all household members.

*TAFE...because there’s a captive audience there, they have to turn up between 9.30[a.m.] to 2[p.m.] or whatever the time is that they’ve got to be there* (service provider)

*An easier way, would be if everything was … translated message for us. Or… talk to us directly [e.g., at TAFE]* (community member)

## Discussion

This exploration found that the Ezidi refugee community in Armidale experience challenges in accessing and using available COVID-19 information. Although the Ezidi are a minority and very specific refugee population in Australia, lessons about their relationship with COVID-19 messaging may overlap with other refugee groups.

We found that the Ezidi people experienced language and health literacy challenges- a situation common to refugee communities, especially those with a long history of displacement [15]. People from non-English speaking backgrounds have trouble on a daily basis, making seemingly simple tasks overwhelming and complicated [6]. For refugees, a background of disjointed education confers low literacy even in their own language, making it difficult to find information and understand government messaging about COVID-19 [15]. We also found that the Ezidi people, in particular the elderly, experienced low digital literacy. The term ‘eHealth literacy’, describes the ability to seek, locate and meaningfully use health information from digital platforms [23]. It has been suggested that simultaneous ability in many learning domains is necessary to provide satisfactory capacity in eHealth literacy. These domains include traditional literacy and numeracy, proficiency with technology and media, and a base level understanding in health and science [23]. Given these pre-existing barriers for many refugees, it is not surprising that health as well as digital illiteracy, at the time of this global pandemic, appears to be a significant factor in reinforcing disadvantages.

We found that the cognitive effects of trauma influenced community’s ability to process health information. Due to the consequence of forced displacement and trauma, refugees have a higher prevalence of mental health problems, such as depression, post-traumatic stress disorder (PTSD) and anxiety compared with other migrants in resettlement countries [24, 25].

Experiences with generational persecution make the Ezidi a particularly vulnerable population regarding psychological distress. PTSD can significantly affect neurocognitive function and produce changes to the amygdala and prefrontal cortex as seen on functional neuroimaging [26]. Fear conditioning related to the PTSD experience was evident in our participants, who used cultural ways of responding to fear, for example, by locking doors and wearing red and white threads. People with PTSD are more likely to have difficulties with attention, working memory and distorted learning capacity, making it difficult to uptake new information [26, 27]. Given the frequency of message change with evolving COVID-19 information, it can be anticipated that those with PTSD might struggle to capture relevant information at appropriate times, even with seemingly adequate delivery of communiqués.

We found that the level of trust towards public figures and governmental messages influenced the access and use of COVID-19 information in the community. The role of public trust and political confidence in healthcare is well described with its importance corroborated in previous infectious outbreaks such as Ebola (2018) and SARS (2003) [24] Public health measures rely upon the engagement of affected individuals and communities to reduce risk. Refugee populations often lack trust in authorities and government systems that have not reliably met their needs [24].

The close-knit nature of the Ezidi community emphasises the crucial role trust plays in acceptance of COVID-19 health messages. This reality corroborates emerging recommendations for the general CALD community [14, 16]. Early harnessing and support of the trusted service providers, who already have the community’s confidence appears to be a logical and effective initiative for sharing government endorsed COVID-19 messages in Australia. By engaging and including key respected community members, tailored approaches to public health education will help build trust with communities [28].

Participants identified ways in which government-driven COVID-19 messages can be improved, in respect to distribution and appropriateness of content. Universal principles, such as provision of messages in language by an audio-visual medium, simplification of content and delivery by trusted service providers are not dissimilar to recommendations for general CALD populations [14–16]. The Ezidi community requires further tailoring of government COVID-19 messages, by considering socio-cultural norms such as age, gender and past history of trauma and torture. Audio links must be culturally appropriate and in language, accessible via preferred and familiar social media platforms. In addition, the content must be seen as endorsed by known and trusted service providers to the Ezidi community. Governments should provide a variety of ways to communicate official messages to communities, accounting for diversity in educational background, eHealth literacy and English proficiency. Learnings from this work with the Ezidi may assist health services direct communication with other diverse communities. By listening and connecting with community members, governments can provide meaningful messages in ways that community members connect with.

We noted different viewpoints between service providers and the Ezidi people, particularly in relation to opinions regarding current roles of influential people in the community, and meanings of certain behaviours observed in the community. This highlights that to understand a health issue within a community one needs to consult widely with different members of that community and other stakeholders. Public health responses need to be informed by such wide consultation.

There are a number of limitations of this study that should be noted when considering the findings. It would have been beneficial to interview more community members; in particular a more heterogeneous groups based on gender, age, level of education, employment level. This is particularly relevant given the diversity of sects within the Ezidi community, even in Armidale. Moreover, comparing Ezidi community with other refugee communities would have provided insights on the similarities and differences with other refugee communities. In addition, it is possible that other Ezidi community members in Armidale use different communication methods, which were not detected in this research.

We were, however, able to gather valuable insights from a unique group of Ezidi community members in Armidale. As with any qualitative study, these findings are not purported to represent the broader population. This research is being expanded to investigate other refugee communities in the Hunter New England region.

## Conclusions

This research provides rich and rare insights from the Ezidi people and their service providers in Armidale NSW; their challenges in accessing government COVID-19 information and suggestions for improvements. Pre-existing structural inequities have been aggravated by the COVID-19 pandemic, with refugees experiencing barriers to accessing healthcare information even before the outbreak of COVID-19. The research found that the experience of being a refugee and consequent health inequities had an influence on how COVID-19 messages were communicated. Furthermore, factors such as literacy, trust and socio-cultural norms are significant factors, which need to be addressed in order to improve communication about COVID-19. COVID-19 information should be tailored to meet the needs of the Ezidi group, as our study shows important disparities in receiving COVID-19 related information, thereby potentially putting this disadvantaged group at greater risk. It is vital that health advice reaches CALD groups in an appropriate and meaningful way. Partnerships and collaboration with Ezidi communities and trusted service providers will enable more positive communication strategies and appears to be a logical step for government health services to employ. Findings from this research have the propensity to benefit other refugee communities facing similar challenges in Australia.

### Supplementary Information

Additional file1 (DOCX 44 kb)

## Data Availability

All data analysed during this study are included in this published article.

## Appendix

**Table:**

Interview questions for community members	
1	Where and how have you been getting your information about COVID-19?
2.	What makes it easy for you to get information from the above sources?
3.	What things make it difficult for you to get information about COVID-19?
4.	What are some ways in which communication about COVID-19 can be improved for you and your community?
5.	Is there anything else you would like to share about how you or your community members get information about COVID-19?

## Abbreviations

CALD: Culturally and Linguistically Diverse
COVID-19: Coronavirus Disease of 2019
NSW: New South Wales
TAFE: Technical and Future Education
SARS: Severe Acute Respiratory Syndrome

## References

[1] Goldin I and M. R., COVID-19 is increasing multiple kinds of inequality. Here’s what we can do about it 2020, World Economic Forum.

[2] Marmot M, et al., Closing the gap in a generation: health equity through action on the social determinants of health. 2088, Lancet. p. 1661–9. 10.1016/S0140-6736(08)61690-6 18994664

[3] Suarez-Balcazar Y, Francisco V.T, Rubén Chávez N (2020). Applying Community-Based Participatory Approaches to Addressing Health Disparities and Promoting Health Equity. Am J Community Psychol.

[4] Braveman P (2011). Health disparities and health equity: the issue is justice. American journal of public health.

[5] Smith MJ (2015). Health Equity in Public Health: Clarifying our Commitment. Public Health Ethics.

[6] Refugee Council of Australia, *Settling in Australia: The challenges* 2019: Surry Hills, Australia.

[7] Reed HE, Barbosa GY (2017). Investigating the Refugee Health Disadvantage Among the U.S Immigrant Population. Journal of Immigrant & Refugee Studies.

[8] Crawley H., Why equality matters if we want to harness the benefits of migration. . 2019, Migration for development and equality: Coventry, UK.

[9] Júnior JG (2020). A crisis within the crisis: The mental health situation of refugees in the world during the 2019 coronavirus (2019-nCoV) outbreak. Psychiatry Res.

[10] Endale T, Jean NSt, Birman D (2020). COVID-19 and refugee and immigrant youth: A community-based mental health perspective. Psychological Trauma Theory, Research, Practice, and Policy.

[11] Brickhill-Atkinson M, Hauck FR (2021). Impact of COVID-19 on Resettled Refugees. Prim Care.

[12] Sheikh-Mohammed M (2006). Barriers to access to health care for newly resettled sub-Saharan refugees in Australia. Med J Aust.

[13] Riggs E (2012). Accessing maternal and child health services in Melbourne, Australia: Reflections from refugee families and service providers. BMC Health Serv Res.

[14] Wild A (2021). Communicating COVID-19 health information to culturally and linguistically diverse communities: insights from a participatory research collaboration. Public Health Research & Practice.

[15] McCaffery KJ (2020). Health literacy and disparities in COVID-19–related knowledge, attitudes beliefs and behaviours in Australia. Public Health Research Practice.

[16] Seale H, et al., Enhancing and supporting the COVID-19 vaccination program- focusing on Culturally and Linguistically Diverse Communities 2021, University of New South Wales

[17] Settlement Services International, All in for Armidale: A whole-of-community approach to Ezidi settlement. (unknown).

[18] Watts S, McMahon T, and S. T., Monitoring community attitudes toward refugee settlement in Armidale, NSW. 2019, University of New England/ Settlement Services International.

[19] NSW Health, Transcultural mental health centre. Yazidi Community (unknown).

[20] Cetorelli V (2017). Mortality and kidnapping estimates for the Yazidi population in the area of Mount Sinjar, Iraq, in August 2014: A retrospective household survey. PLoS Med.

[21] Tolley, E., et al., Qualitative Methods in Public Health: A Field Guide for Applied Research, 2nd Edition, ed. E. Tolley, et al. 2016, San Francisco, CA: Wiley: Jossey-Bass.

[22] Carter SM, Ritchie JE, P Sainsbury (2009). Doing good qualitative research in public health: not as easy as it looks. NSW public health bulletin.

[23] Norman CD, Skinner HA (2006). eHealth Literacy: Essential Skills for Consumer Health in a Networked World. J Med Internet Res.

[24] Chen W (2017). Pre-migration and post-migration factors associated with mental health in humanitarian migrants in Australia and the moderation effect of post-migration stressors: findings from the first wave data of the BNLA cohort study. Lancet Psychiatry.

[25] Heeren M (2014). Psychopathology and resident status - comparing asylum seekers, refugees, illegal migrants, labor migrants, and residents. Compr Psychiatry.

[26] Hayes JP, Vanelzakker MB, Shin LM (2012). Emotion and cognition interactions in PTSD: a review of neurocognitive and neuroimaging studies. Front Integr Neurosci.

[27] McNally RJ (2006). Cognitive abnormalities in post-traumatic stress disorder. Trends Cogn Sci.

[28] World Health Organization, TIP Tailoring Immunization Programmes 2019, World Health Organization, Regional Office for Europe.

