# Supplementary material for: Ezidi voices: The communication of COVID-19 information amongst a refugee community in rural Australia- a qualitative study
Source: Int J Equity Health. 2022 Jan 21;21:10. doi: 10.1186/s12939-022-01618-3 (PMC8777446; doi:10.1186/s12939-022-01618-3)
Supplement: Supplementary file 1 — Additional file1 (DOCX 44 kb) [file 12939_2022_1618_MOESM1_ESM.docx]

**RESPONSE TO REVIEWERS’ COMMENTS**

Dear IJEH Editor,

Thank you for considering our paper, entitled “Ezidi voices: The communication of COVID-19 information amongst a refugee community in rural Australia- a qualitative study” for publication. We appreciate both reviewers’ time and thoughtful feedback. We have addressed reviewers’ comments point by point in text below and have made relevant track changes in the body of the latest version manuscript.

We believe that the suggested modifications have strengthened our paper and trust that our responses will satisfy you and the reviewers.

Kind regards,

Sunita Rebecca Healey and co-authors 17/12/21

|  | **Reviewers’ comments** | | **Authors’ response** | **Line number or relevant section** | **Relevant excerpts of the paper, additions highlighted in yellow, reduction in strikethrough** |
| --- | --- | --- | --- | --- | --- |
|  | **Reviewer #1 (18 comments)** | |  |  |  |
| 1 | General comment | This is an extremely relevant and interesting study of COVID experiences in a minority community. This paper uniquely describes how the Ezidi community's past experiences shaped their response to COVID, and thus their needs. It is well done and deserves to be published, although I recommend some revisions. | We thank the reviewer for a positive appraisal of our paper | N/A | N/A |
| 2 | Abstract | Hyphenate "government led" in the abstract - it is a compound adjective | Corrected, with thanks | line 70  (Abstract>  Background) | … and how the government-led COVID-19 information communication could be improved. |
| 3 |  | Use lower case for "cultural" in the phrase "…messages; Cultural, social and gender norms…" | Corrected, with thanks | Line 79  (Abstract> Results) | …messages; ~~C~~cultural, social and gender norms |
| 4 |  | Use either "…a community's uptake" or "communities' uptake" | Corrected, with thanks | Line 80  (Abstract> Results) | …service providers are key in ~~community’s~~ communities’ uptake of COVID-19 messages… |
| 5 |  | "and-message delivery" should not be hyphenated | Corrected, with thanks | Line 83  (Abstract> Results) | …communiqués ~~and-message~~ and message delivery… |
| 6 | Background | Could the authors provide a sentence or two about the social system of the Ezidi? For example, whether they are historically patriarchal, democratic, etc.? I am curious how this social system is reflected in the participants ("influential…men" and "Ezidi women"). | We have now included information which expands on the social system of the Ezidi. The mention of holy men points to the importance of males in the storytelling culture. | Lines 127-131  (Background) | Ezidi society follows a hierarchical caste system and marriages are required to remain within the caste [19]. The Ezidi have strong oral traditions, passing on their customs through generations of storytelling in the Kurmanji dialect of the Kurdish language, especially through holy men [17, 19]. |
| 7 |  | "…people of colour have and continue to experience…" should be "…people of colour have experienced and continue to experience" | Corrected, with thanks | Line 102  (Background) | …people of colour have experienced and continue to experience…. |
| 8 |  | "trickledown" should be "trickle-down" | Corrected, with thanks | Line 116  (Background) | … a trickle-down effect… |
| 9 | Research design | "comprising of" should just be "comprising" or "comprised of" | Corrected, with thanks | Line 154  (Methods> Research design) | … by a multidisciplinary research team comprised ~~comprising~~ of representatives from Multicultural Health, |
| 10 | Participants | In the phrase "2) Influential Ezidi community members" the word influential should not be capitalized | Corrected, with thanks | Line 163  (Methods> participants) | …2) ~~I~~influential Ezidi community members |
| 11 | Procedure | The second paragraph should begin "A semi-structured interview guide…" | Corrected, with thanks. We have further corrected this line by using a more active tone in the sentence structure. | Line 187  (Methods> procedure) | We developed a s~~S~~emi-structured interview guide ~~was developed~~ and piloted it with a few Syrian… |
| 12 |  | Would the authors please define "HNE" at first use | Corrected, with thanks. Additional correction has been made to the grammatical tense. | Lines 193-195  (Methods> procedure) | ~~Focus groups~~ The interviewers were ~~facilitated by~~ employees of the Hunter New England (HNE) Multicultural Health Services: two refugee health nurses and a multicultural health liaison officer. One of the nurses was ~~is~~ well known and accepted by the community. |
| 13 | Results | It would be useful to have a general description of (1) how the Ezidi responded to COVID (besides locking their doors) and (2) how badly COVID affected their community. Did the Ezidi know to stop social events and gatherings and wear masks? Generally, were a lot of Ezidi infected compared to the Australian population? When the vaccines became available, did the Ezidi get them? (There is one quote about mask information from a service provider, but it would be useful to know whether this confusion affected mask wearing in the community.) I'm sure that detailed information is out of scope for this paper but a general description would help with understanding vulnerability to infection. | We thank the reviewer in their interest. We have now included additional information in ‘Setting’ to clearly state that there was no community transmission of COVID-19 at the time of the study.  Although we did retrieve information about the community’s perception about the vaccine, we felt it didn’t fall into the scope of the study (which focussed on communication). It is therefore not included within this manuscript.  Hunter New England Health provided a nurse-led vaccination clinic to the Ezidi community, many months after the completion of our study. The refugee health nurse vaccinated community members, with interpreters available, and there was good uptake of this service. This was reportedly due to the good relationship between community members and refugee health nurse. | Line 170-175  (Methods> setting) | During the study period many countries were experiencing significant waves of infection from new variants. Although there were no COVID-19 transmissions amongst the Ezidi in Armidale, the community were aware of the effects of COVID-19 affecting Ezidi family and friends overseas. The roll out of the vaccine program had commenced but only a small proportion of people were fully vaccinated. |
| 14 |  | Page 11, there should be a comma after the word therefore (…technology; therefore they relied…) | Corrected, with thanks | Line 241  (Results> theme 1) | … technology; therefore, they relied on younger members… |
| 15 |  | Page 12, would you please explain what you mean by "…younger men may have desirable skills such as bilingual ability and employment…" - employment being a skill? | Thank you for noting the confusion in our original wording. The authors have now changed the term and explained better. We have also extended the quote provided by an influential member, to improve understanding | Line 273-276  (Results> theme 2) | … although younger men may have desirable ~~skills~~ attributes such as bilingual ability and ~~employment~~ networking capability through workplaces, service providers felt that they were hesitant to pass on information without authorisation by respected older males~~.~~: |
|  |  |  |  | Line 519  (Results> theme 5) | … educated and ~~employed~~ connected to people outside the Ezidi community through workplaces. |
|  |  |  |  | Line 290-293  (Results> theme 2) | *They [community members are] usually asking people who are working already with the organisation.... we have got 2 or 3 people who are working with them. So, they ask them. If there is any situation happen in Armidale in particular, we all would be connected with each other* (influential member) |
| 16 |  | Page 18, "under supported" should be hyphenated | Corrected, with thanks | Line 420  (Results> theme 4) | … reported feeling under-supported by government… |
| 17 |  | Page 23, please define "TAFE" at first use | Thank you for bringing this to our attention. We note that ‘TAFE’ is in fact used earlier than the reviewer mentioned. We have corrected it in first use and added a brief explanation that TAFE is a tertiary education setting. The acronym ‘TAFE’ is used for the remainder of document | Line 370-371  (Results> theme 3) | as schools, a tertiary education setting (Technical and Further Education- TAFE), ~~TAFE (Technical an Further Education)~~ settlement |
| 18 | Conclusion | Could the authors provide a concise list of how to create healthcare information for CALD populations in general besides the Ezidi? From the paper, I gather that videos are better than text/images, trusted partners should deliver the information, the information should be consistent, etc. A short list like this (or even a figure or table) would be very helpful for putting the results of this paper into practice. | Thank you for this recommendation.  We agree that a table will best demonstrate the information in a concise way. Therefore, a table has been included in results, which documents the improvements suggested by the study participants. The table is specific to the Ezidi group in the study.  As the CALD community is not homogenous and may be made up of many other groups (e.g., migrant workers, international visitors etc.) the authors are hesitant to overstate our results for all CALD populations. Hence, we have not created a table in ‘Discussion’ for the general CALD community. | Line 469  (Results> theme 5) | …format and delivery of COVID-19 information could be improved to better reach the Ezidi community (Table 1). |
|  |  |  |  | Line 548-550  (Results> theme 5) | Table 1: Suggestions for improvement of official COVID-19 information for Ezidi community in Armidale, Australia (2021) **Please see the manuscript directly to view the table* |
|  |  |  |  | Lines 616-623  (Discussion) | Governments should provide a variety of ways to communicate official messages to communities, accounting for diversity in educational background, eHealth literacy and English proficiency.  Learnings from this work with the Ezidi may assist health services direct communication with other diverse communities. By listening and connecting with community members, governments can provide meaningful messages in ways that community members connect with. |
|  | **Reviewer #2: (9 comments)** | | **Authors’ response** |  |  |
| 1 | General comment | The article offers important insights into formulating and delivering health messaging to CALD. This is ever more important in the backdrop of rapid social changes and geographical movements of people. There are a few areas that the authors could consider addressing to improve the paper: | We thank the reviewer for a positive appraisal of our paper. | N/A | N/A |
| 2 | Major issue: Method 1) | In the abstract, there is a mention of a participatory framework. The title also mentions that it is a participatory study. However, the main method section does not mention or refer to any such framework. It would be helpful if the authors clearly mention what framework was used and how the framework guided their study. It is also important to note that just interviewing community groups does not constitute a participatory study. Participatory studies have specific definitions and methods, especially how community groups are engaged in designing a study and taking action following from the recommendations of the findings. | We thank the reviewer for their comment. Although we used elements of the participatory approach (i.e., interviewers were connected to the community and the interpreters had intel to the community), we did not use all elements of a participatory approach, or Participatory Action Research. Therefore, we removed mention of ‘participatory’ from the paper and replaced the term with ‘qualitative study’ in the title. | Line 3  (Title page) | **Ezidi voices: The communication of COVID-19 information amongst a refugee community in rural Australia- a ~~participatory~~ qualitative study.** |
|  |  |  |  | Line 56  (Title) | **Ezidi voices: The communication of COVID-19 information amongst a refugee community in rural Australia- a ~~participatory~~ qualitative study.** |
|  |  |  |  | Line 73-74  (Abstract> Methods) | … COVID-19 messaging. ~~Working within a participatory framework the~~ Multicultural and Refugee Health… |
| 3 | Major issue: Method 2) | Word restriction may have led to curtailing details of the methods undertaken but some key information need to be there for the readers to know how the study was conducted. Please consider clarifying-  a.      Data collection method: interview or FGD? b.      if FGD, how many groups consisting of how many participants? c.       Were community leaders and women all in the same discussion group? Why or why not? d.      Language FGD/interview conducted in? e.      Transcripts needed translation? by whom? | Thank you for the advice to expand on methodology. Three focus groups were conducted. Later, we individually interviewed (using the same guide) an additional influential member.  We have now added a paragraph detailing the data collection method, including the number of participants and composition details, language and transcription.  As the interpreter translated during the interview, the non-English components of the audio were not transcribed nor translated. Only the English component (as provided by the interpreter or directly by participants) was transcribed. | Line 119-210  (Methods> procedure) | We conducted three focus group discussions in English: service providers (four), influential community members (six) and community members (three). To minimise power imbalance, we interviewed the influential community members in a separate group to the community members. Kurdish-Kurmanji health interpreters were available on-site to translate discussions with community members.  Seeking richer data and clarification of cultural matters, researchers interviewed an additional (English speaking) influential community member by teleconference, approximately three months after the initial focus group discussions.~~Group~~ Focus group discussions ~~discussions~~ lasted 30-60 minutes and were audio-recorded. A native English-speaking member of the research team transcribed verbatim the English components of the collected data. ~~and transcribed verbatim. Kurdish Kurmanji health interpreters were provided on site for discussions with community members.~~ |
|  |  |  | To assist with reduction of the word count, we have removed duplicated information at the start of results. | Line 223-224  (Results) | ~~Initial interviews and focus groups were conducted with four service providers, seven Ezidi men and three Ezidi women.~~ |
| 4 | Major issue: Method 3) | Why verbal consents were obtained from the community participants? Good to be explicit about the reasons. | Thank you for your interest in the consent process. We have now included additional information about the participants’ hesitancy in documenting a signature. At the time, the researchers felt it was unsuitable to probe into the reasons for refusing to sign, as we did not want participants to feel any further discomfort with the process | Lines 178-184  (Methods> procedure) | The p~~P~~articipant information statement for community members was presented as a short video delivered in Kurdish-Kurmanji language prior to obtaining consent. ~~Prior to obtaining verbal consent, c~~ Community members had an opportunity to ask questions about the study, with an on-site Kurdish-Kurmanji interpreter present. Verbal consent was collected, as some community members refused to document a signature on the consent form. Service providers received written information statement and provided a written consent. Interviewers sensed discomfort from the community members and did not probe further for signatures. |
| 5 | Major issue: Results 1) | Gender has been identified as a factor in how people received and responded to COVID-19 health messaging. Yet there has been no effort to show what the women participant themselves shared about their experiences and their own barriers. It would be helpful to how women participants' views agreed/differed from community leaders'. | Thank you for this suggestion.  We have now added a paragraph with some insights given by both male and female participants about their different responses.  As we have small numbers, generalisations about gender cannot be drawn from our data. However, as service providers had reported observing gender as a factor in people’s response to COVID-19 health messaging, it remains included in the participant recommendation section. | Lines 307-315  (Results> theme 2) | One female community member reported that she relied on male relatives (uncles and brother) to gather information about COVID-19, as they were literate in Arabic. Another female community member was literate herself and said she actively sought out COVID-19 information. An older male influential member talked about the variable community uptake of messages he and other elders had shared amongst the community through word of mouth. Some older males reported that despite literacy in Arabic, being digitally illiterate and having low proficiency in English meant that accessing COVID-19 information remained difficult. |
| 6 | Major issue: Discussion 1) | A key barrier for the elderly community members was digital illiteracy. Yet one of the recommendations is messaging through FB and WhatsApp. Some discussion around this divergency would be interesting and how this would be taken up on the ground. | Thank you for this comment.  Although social media is a recommended platform, it is certainly not the only method which should be used to share information. Diversity amongst a community precludes that there should be diverse responses by government. We have made comment to this effect in Discussion and within a newly fashioned table in Results. | Line 616-623  (Discussion) | Governments should provide a variety of ways to communicate official messages to communities, accounting for diversity in educational background, eHealth literacy and English proficiency.  Learnings from this work with the Ezidi may assist health services direct communication with other diverse communities. By listening and connecting with community members, governments can provide meaningful messages in ways that community members connect with. |
|  |  |  |  | Line 548-550  (Results-table) | *In table:* ‘Recognise diversity within the ethnic group’…… ‘Choose multiple methods to distribute official information’ |
| 7 | Minor issue 1) | In line 60 and a few other places the authors wrote, "people who were refugees", probably indicating a resettled community. However, in line 142, they are referred to as a refuge community. Please clarify who are the study target and population groups. | Thank you for noting this inconsistency.  Although the Ezidi community in Armidale are a resettled community, their refugee background and recency of migration is significant. Therefore, to be consistent with the remainder of the document, we have adopted the term ‘refugees’ in replacement of ‘people who were refugees’. | Line 61  (Abstract> background) | ~~People who were refugees~~ Refugees are a unique sub-set of the CALD communities and are… |
|  |  |  |  | Line 106  (Background) | ~~People who were refugees~~ Refugees are a unique sub-set of culturally and linguistically diverse (CALD) communities… |
|  |  |  |  | Line 108  (Background) | ~~Resettled people who were r~~Refugees are at a significant health disadvantage compared with non-refugee |
| 8 | Minor issue 2) | It is not clear if this is because their refugee status has changed since the study or for some other reason. Isn't the intention of the paper to specifically share perceptions and experiences of a refugee population? | Thank you for seeking clarification about this issue. As the reviewer mentions, our intention of the paper is to specifically share perceptions of a refugee population.  The technicalities of the term ‘refugee’ has been debated amongst our research team, as the community participants are no longer fleeing danger from their homeland upon arrival in Australia. Upon arrival in Australia, people with refugee visas become permanent residents.  For simplicity and universal understanding (including search engines), we have left the term ‘refugee’ to describe the group of our participants, with explanation that they are resettled people. | Line 64-65  (Abstract> background) | The recently resettled Ezidi refugee community in a regional area of Australia is an example… |
|  |  |  |  | Line 125  (Background) | …home to various groups of resettled people including approximately 600 Ezidi. |
| 9 | Minor issue 3) | Some of the quotes have incomplete sentences and bad translation making them difficult to comprehend fully. Some editing will help these quotes. | Thank you for your insights regarding the clarity of quotes.  We have reviewed each quote and edited as appropriate. | Lines 259, 264, 267, 292-293, 325-326, 336, 384-385, 392, 398, 402, 424, 427, 445, 450, 509-510, 541, 544-546  (Results) | Multiple edits to quotes in text throughout the Results section. Please see the manuscript directly. |
|  | **Additional author-led improvements to revised manuscript**  **(5 comments)** | | | **Line number or relevant section** | **Relevant excerpts of the paper, additions highlighted in yellow, reduction in strikethrough** |
| 1 | Extra citing inserted to reference approach/thematic analysis. Reference has previously been cited in manuscript | | | Lines 214-217  (Methods> procedure) | Paired analysis involved discussing and comparing individual analyses and creating a combined analysis file for each transcript [21]. After reviewing several transcripts, similar concepts were grouped into categories [21]. |
| 2 | Rephrasing to reduce word count | | | Lines 211-214  (Methods> procedure) | Senior qualitative researchers provided guidance and oversight for data analysis ~~and rigor, while individual interviewers conducted paired analyses~~. Each transcript was read and reviewed separately by two ~~researchers~~ interviewers, each creating an individual analysis file before meeting to pair their analyses. |
| 3 | Updated email address for author #3 | | | Line 24  (Title page) | [~~Peter.Massey@health.nsw.gov.au~~](mailto:Peter.Massey@health.nsw.gov.au)  Peter.Massey@jcu.edu.au |
| 4 | Updated details for author #7 | | | Line 48-52  (Title page) | ~~Research Fellow - Health Protection~~  ~~HNE Population Health Unit~~  ~~Booth Building, Wallsend Health Services~~  ~~Longworth Ave, Wallsend NSW 2287~~  Research Fellow  The University of Newcastle  University Drive, Callaghan NSW 2308, Australia |
| 5 | Addition of Country details for authors #1 and #2 | | | Lines 10, 17  (Title page) | Australia |
